# *In silico* analysis identified bZIP transcription factors genes responsive to abiotic stress in Alfalfa (*Medicago sativa* L.)

**DOI:** 10.1186/s12864-024-10277-3

**Published:** 2024-05-21

**Authors:** Atit Parajuli, Bhabesh Borphukan, Karen A. Sanguinet, Zhiwu Zhang

**Affiliations:** Department of Crop and Soil Science, Washington State University, 99164 Pullman, WA USA

**Keywords:** Alfalfa, bZIP transcription factor, Phylogenetic analysis, Expression pattern, Abiotic stress

## Abstract

**Background:**

Alfalfa (*Medicago sativa* L.) is the most cultivated forage legume around the world. Under a variety of growing conditions, forage yield in alfalfa is stymied by biotic and abiotic stresses including heat, salt, drought, and disease. Given the sessile nature of plants, they use strategies including, but not limited to, differential gene expression to respond to environmental cues. Transcription factors control the expression of genes that contribute to or enable tolerance and survival during periods of stress. Basic-leucine zipper (bZIP) transcription factors have been demonstrated to play a critical role in regulating plant growth and development as well as mediate the responses to abiotic stress in several species, including *Arabidopsis thaliana*, *Oryza sativa, Lotus japonicus* and *Medicago truncatula*. However, there is little information about bZIP transcription factors in cultivated alfalfa.

**Result:**

In the present study, 237 bZIP genes were identified in alfalfa from publicly available sequencing data. Multiple sequence alignments showed the presence of intact bZIP motifs in the identified sequences. Based on previous phylogenetic analyses in *A. thaliana*, alfalfa bZIPs were similarly divided and fell into 10 groups. The physico-chemical properties, motif analysis and phylogenetic study of the alfalfa bZIPs revealed high specificity within groups. The differential expression of alfalfa bZIPs in a suite of tissues indicates that bZIP genes are specifically expressed at different developmental stages in alfalfa. Similarly, expression analysis in response to ABA, cold, drought and salt stresses, indicates that a subset of bZIP genes are also differentially expressed and likely play a role in abiotic stress signaling and/or tolerance. RT-qPCR analysis on selected genes further verified these differential expression patterns.

**Conclusions:**

Taken together, this work provides a framework for the future study of bZIPs in alfalfa and presents candidate bZIPs involved in stress-response signaling.

**Supplementary Information:**

The online version contains supplementary material available at 10.1186/s12864-024-10277-3.

## Introduction

Alfalfa (*M. sativa* L.) is a highly outcrossing forage legume, widely cultivated in the United States with approximately 16 million hectares [1]. It is well suited for animal and livestock feed due to its high nutritional content. It also improves soil fertility through its symbiotic association with the soil bacterium *Sinorhizobium meliloti* for biological nitrogen fixation, which augments the nitrogen content in the soil for future crops [2–4]. This deep-rooted perennial crop also helps to prevent soil erosion. However, genetic improvement in terms of forage yield has been relatively stagnant in alfalfa [1]. Major hinderances are genomic complexity, severe inbreeding depression upon selfing, and self-incompatibility which complicate alfalfa breeding. Although the multi-purpose use of alfalfa increases its demand, adverse environmental conditions result in abiotic stresses, and ultimately hamper production. Breeding for stress resistance improves production to some extent; however, lack of completely annotated genome and expression profile data, eventually creates knowledge gap in fully understanding genotype and phenotype associations for stress-related traits.

The sessile nature of plants inevitably exposes them to adverse environmental conditions such as abiotic stress. However, plants have developed diverse mechanisms to cope with these abiotic stresses. One of them is the synthesis of proteins, metabolites, and other compounds to aid in survival through abiotic stress, which is often controlled by transcription factors (TFs). Transcription factors play a critical role in responses to environmental stresses via binding to cis-regulatory elements in promoters to regulate downstream gene expression. In plants, approximately 7% of the genome codes for transcriptional regulators, which bind promoter elements of downstream genes through their conserved sequence-specific DNA-binding domain [5]. Among the 64 families [6] of transcription factors identified in the plant kingdom, the bZIP (basic leucine zipper) family is one of the largest and most diverse [5–7].

The basic leucine zipper (bZIP) family is distinguished by its highly conserved bZIP domain composed of 60–80 amino acids [7]. Structurally, the bZIP domain is divided into two functionally distinct regions: a basic region and a leucine zipper motif [7]. The basic region is composed of an invariant motif (N-x7-R/K-x9) of 18 amino acids residues that facilitates sequence-specific DNA binding, while the leucine zipper contains several heptad repeats of leucine or other bulky hydrophobic amino acids such as isoleucine, valine, phenylalanine, or methionine, for dimerization specificity [6–8]. Molecular studies of bZIP genes in *A. thaliana* show that they are involved in the regulation of diverse biological processes including pathogen defense, light and stress signaling, seed maturation, and flower development [8]. Additional information on the bZIP transcription factor family has provided evidence of their role in response to biotic and abiotic stresses in a diversity of plant species [8, 9].

The availability of whole-genome sequences for plants allows the identification or prediction of bZIP TF family members at the genome-wide level. The number of bZIP TFs identified in different plant and crop species varies from 78 (*AtbZIPs*) in *A. thaliana* [8, 10], 89 (*OsbZIPs*) in *O. sativa subs. japonica* [7], 125 (*ZmbZIPs*) in *Zea mays* [11], 131 (*GmbZIPs*) in *Glycine max* [12], 92 (*SbbZIPs*) in *Sorghum bicolor* [13], 55 (*VvbZIPs*) in *Vitis vinifera* [14], 64 (*CsbZIPs*) in *Cucumis sativus* [15] and 247 (*BnbZIPs*) in *Brassica napus* [16]. The bZIP transcription factors play crucial roles in developmental processes and environmental tolerance in response to multiple stresses. They are involved in the regulation of the seed development [17, 18], cell elongation [19, 20], vascular development [19], flower development [21–25], somatic embryogenesis [25], as well as in nitrogen/carbon and energy metabolism [26–28].

In addition to functions in plant growth and development, bZIPs also play an important role in responses to abiotic and biotic stresses. Several bZIPs from *A. thaliana* (*AtbZIP17, AtbZIP24, AtbZIP12*), rice (*OsbZIP12, OsbZIP72, OsABF1*), and soybean (*GmbZIP44, GmbZIP62, GmbZIP78*) were found to positively regulate salt stress adaptation in plants either directly or indirectly [12, 29–33]. Several bZIPs from rice (*OsbZIP52/RISBZ5, OsbZIP16, OsbZIP23, OsbZIP45, AREB1, AREB2, ABF3*) were also found to be involved in the drought tolerance [34–37]. *OsbZIP52/RISBZ5* negatively regulates cold stress responses [36] while *OsbZIP72* was a positive regulator of ABA responses [31]. Similarly, overexpression of *GmbZIP44, GmbZIP62*, and *GmbZIP78* reduced ABA sensitivity [12]. Interestingly, group D or so-called TGA bZIPs plant a role in systemic acquired resistance (SAR) and pathogen resistance [38, 39] (Fu et al., 2013; Gatz, 2013). However, there is little published information about the bZIP transcription factor family in cultivated alfalfa and its role in stress resistance.

With the availability of a chromosome-level genome assembly in alfalfa [40], we conducted a genome-wide search to identify and characterize alfalfa bZIP transcription factors. Since bZIP transcription factors were identified to play significant roles in the regulation of the abiotic stress tolerance [10, 11], we speculated various bZIP transcription factors would be differentially expressed throughout distinct developmental stages and in response to abiotic stresses in alfalfa as well. The present study identifies several bZIPs from a protein database in tetraploid alfalfa (*M. sativa*). We also analyzed differential gene expression from transcriptomics during ABA, drought, salt, and cold stress conditions and verified a subset of differentially expressed bZIPs via qRT-PCR. This study will facilitate functional analysis of the bZIP transcription factor family in alfalfa. The identification of functions of alfalfa bZIP transcription factors during abiotic stress conditions will further help breeding efforts for improved stress tolerance.

## Results

### Identification of the alfalfa bZIP gene family

We identified 237 bZIP sequences with the intact bZIP domain in alfalfa (*M. sativa*). These sequences were named *MsbZIP1* to *MsbZIP237* based on the order identified in the protein sequence database [40]. We compared the genome size and number of bZIPs in different models and crop species (Table 1). The comparison shows that alfalfa has the highest number of bZIP sequences. Since the diploid model legume *M. truncatula* with a genome size of 390 Mega Base (Mb) has 75 bZIP sequences, tetraploid alfalfa is expected to have double the number of bZIP sequences. Not surprisingly, the number of bZIP TFs identified in alfalfa was 237, which likely represents the complete number of bZIP for tetraploid alfalfa.

**Table 1:** Comparative genome size and number of bZIP proteins in different model crops used in the study

Species	Chromosome	Genome Size	Number of bZIPs
*A. thaliana* (8, 10)	2n = 2x = 10	135 Mb	78
*B. napus* (16)	2n = 2 × _1_ + 2 × _2_ = 38	1.16 Gb	247
*L. japonicus* (6)	2n = 2x = 12	470 Mb	70
*M. truncatula* (41)	2n = 2x = 16	390 Mb	75
*O. sativa* (7)	2n = 2x = 24	430 Mb	89
*M. sativa* (Current Study)	2n = 4x = 32	3,150 Mb	237

### Chromosomal distribution of bZIP genes

Among the identified 237 bZIP genes, 233 were annotated among 32 chromosomes and the remaining 4 genes were annotated in 6 different Contigs (Contig 37,287, Contig 43,349, Contig 51,828, Contig 57,601, Contig 57,602, Contig 57,603). The largest gene was in Group A with a length of 688 amino acids while the smallest genes were in Group S with 119 amino acids in length (Supplementary Table 1). The distribution of genes on all the 32 chromosomes was also different (Supplementary Fig. 2). In most of the chromosomes, they were distributed throughout, while, in a few of the chromosomes, these genes are concentrated towards telomeric regions of the chromosomes. The chromosomal distribution of bZIP genes and their chromosome related information is provided with supplementary information.

### Phylogenetic analysis and multiple sequence alignment

**Fig. 1:**
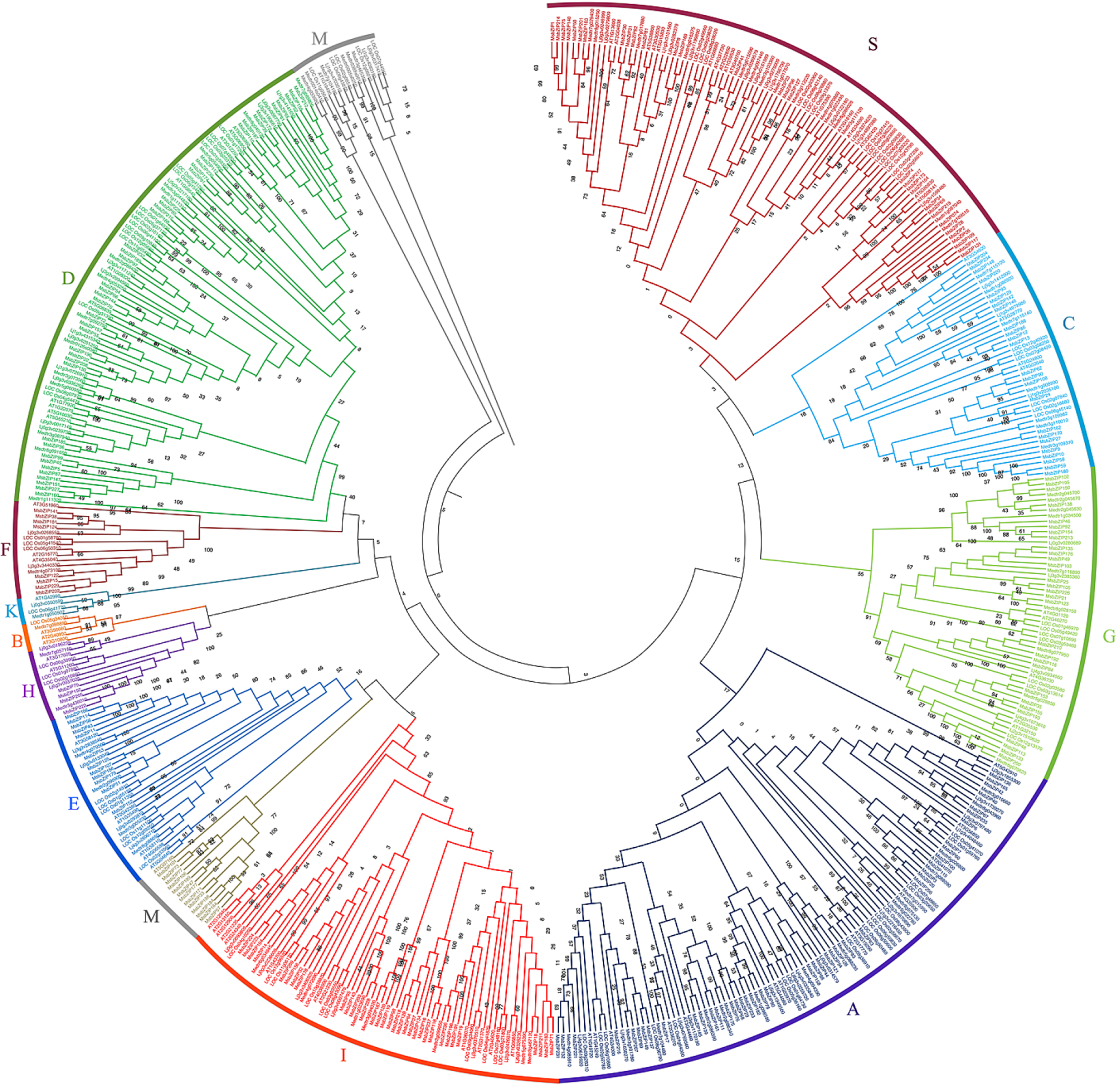
Phylogenetic analysis and group classification of bZIP proteins from alfalfa. 237 bZIP proteins and 312 proteins from *A. thaliana* (78), *L. japonicus* (70), *M. truncatula* [43] and *O. sativa* (89) were used to create a neighbor joining tree with 1000 bootstraps. The bZIPs are grouped into 11 groups (**A - K, M** and **S**) based on tree topology results from *A. thaliana* and *M. truncatula.* A detailed information of the tree including the genes from all the species mentioned above is presented in Genes expressed during stress conditions are distributed throughout the groups. Group D contained genes highly expressed during drought stress conditions and ABA, while Group I and S contained genes that are upregulated during salt stress conditions. Although genes highly expressed during cold stress conditions were distributed over different groups, most of them were from group A and S

**Fig. 2:**
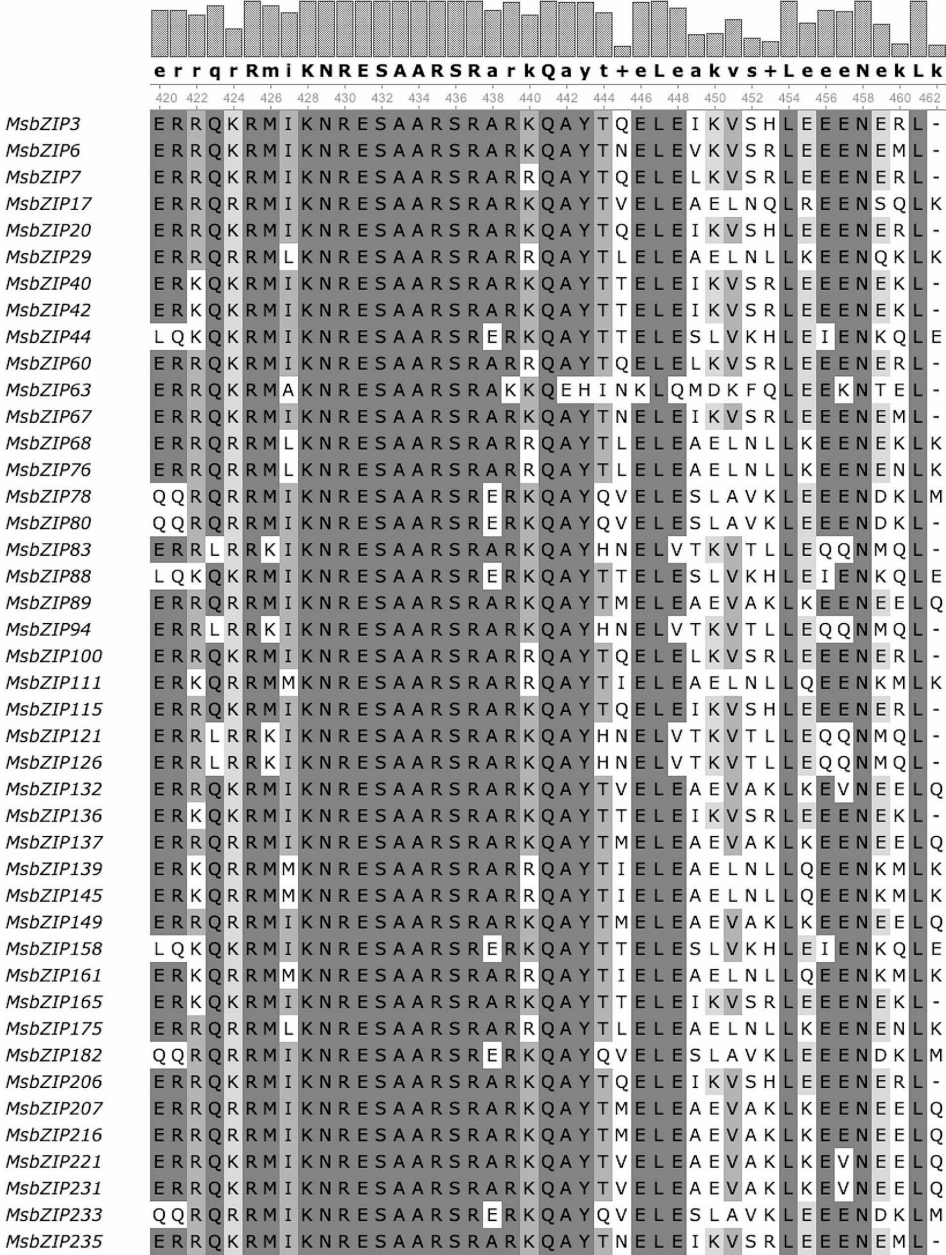
Multiple sequence alignment of alfalfa bZIP proteins of group **A.** The alignment was performed using MUSCLE 3.8.31 and visualized using Unipro UGENE v.33. The A group contains 43 bZIPs in alfalfa, which are highly expressed during abiotic stress. A consensus sequence is provided at the top of the figure (in bold). The bars above the consensus sequence present the percentage of consensus amino acid base between the aligned sequences. The ruler below the consensus sequence provides the position of the amino acid base on the aligned sequences. The color changes from light to dark with dark color indicating highly conserved amino acid bases. The consensus sequence at the top indicates the level of conservation with a capital letter indicating high conservation and a small letter with low conservation. The highly conserved bZIP domain (N-x[7]-[RK]-x[9]-L-x[6]-L-x[6]-L), between 429 to 461, is presented in dark color

The identified 237 bZIP proteins were divided into 10 groups (A, C, D, E, F, G, H, I, M, and S) based on the topology of the tree developed in *Arabidopsis thaliana* [8, 10] and were used to generate a phylogenetic tree along with protein sequences from *A. thaliana*, *L. japonicus*, *M. truncatula* and *O. sativa* (Fig. 1). Alfalfa bZIP proteins fell into 10 different groups and numbers ranged from four (H) to forty-three (A); however, no members were identified for groups B, J and K. To identify common conserved domains amongst the sequences, we carried out multiple sequence alignment. The alignment of 237 bZIP protein sequences showed the presence of intact and highly conserved bZIP domains (N-x [7]-R/K-x [9]-L-x [6]-L-x [6]-L) (Fig. 2, Supplementary Fig. 1). The domain is divided into the basic region with ∼ 18 amino acids residues containing nuclear localization signal followed by an intact N-x [7]-R/K motif while the leucine zipper region contains heptad repeats of leucines or other bulky hydrophobic amino acids with nine amino acids towards the C-terminus [10]. The presence of the intact bZIP domain further validates the identified sequences as bZIP proteins.

### Conserved protein domain analysis

**Fig. 3:**
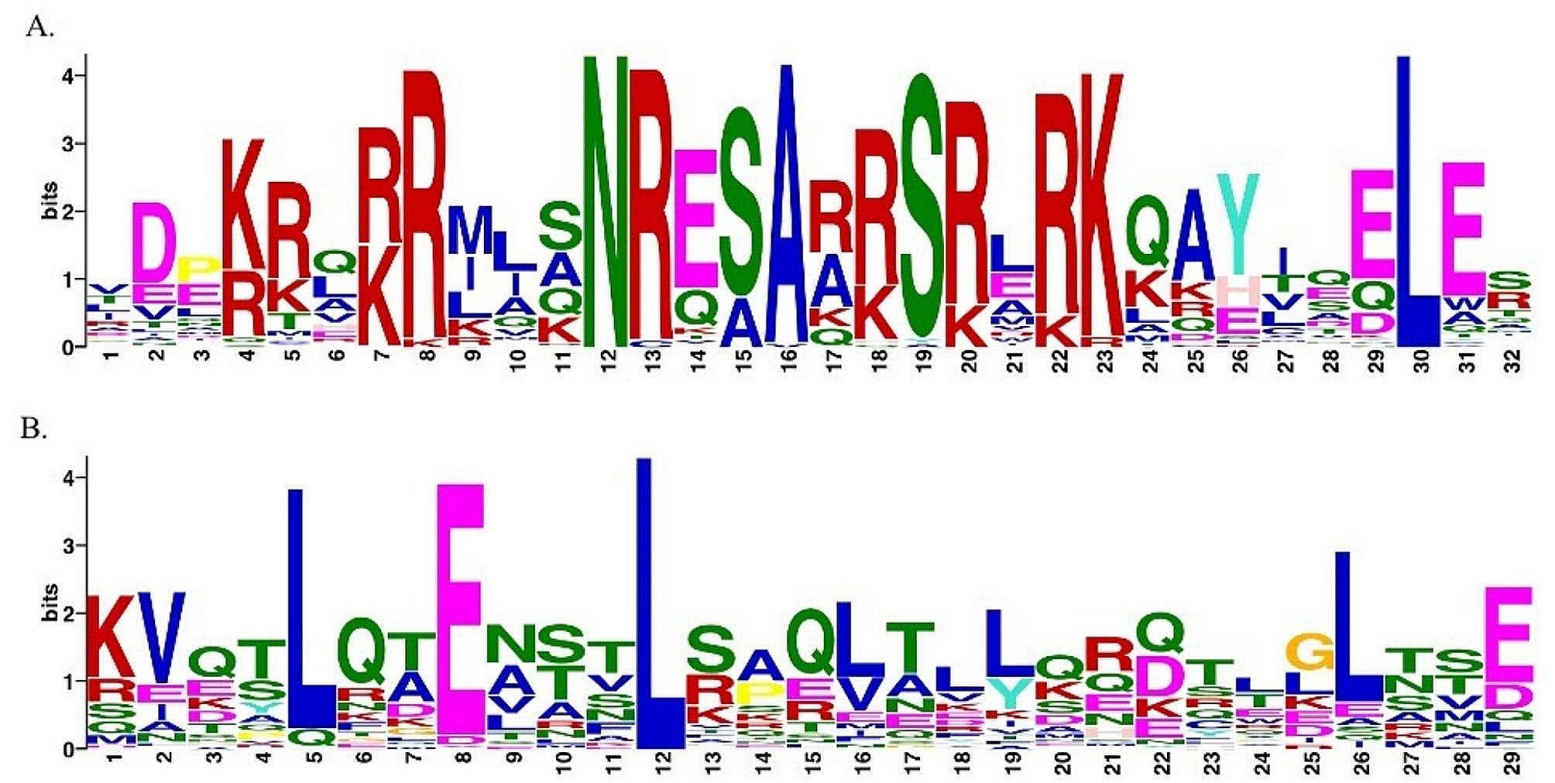
Conserved bZIP domain. (**A**) the conserved basic region of the bZIP motif (motif 12–29). The basic region is composed of an invariant motif (N-x7-R/K-x9) with 18 amino acid residues which can be observed. The basic region facilitates the sequence specific DNA binding. (**B**) The leucine zipper region that contains the heptad repeat of leucine (motif 5–19) or other bulky hydrophobic amino acids is represented by the figure. This region is important as it facilitates the dimerization specificity. This consensus sequence was generated using MEME suite 5.3.3. MEME (Multiple Em for Motif Elicitation) allows discovery of novel motifs in collection of nucleotides or protein sequences. The height of the character corresponds to how frequently the character occurs at that position in the motif

Identification of conserved protein motifs helps to elucidate protein functions and bZIPs usually possess additional conserved motifs that could provide sites for activation [44]. Using the “MEME” (Multiple Em for Motif Elicitation) program [45], 25 conserved motifs were identified in the 237 bZIPs (Supplementary Table 2, Supplementary Fig. 6). The conserved motifs were specific to the different groups identified in this study (Supplementary Fig. 6). Among the identified motifs, the basic region of the bZIP, containing an invariant motif (N-x7-R/K-x9) with 18 amino acid residues was found (Fig. 3A), while the leucine zipper region that contains the heptad repeat of leucine or other bulky hydrophobic amino acids was also identified (Fig. 3B). The basic region facilitates sequence specific DNA binding whereas the leucine zipper region is important for dimerization specificity. However, the function of the 23 motifs that were also identified in the bZIP sequences are unknown and require further study.

### In silico functional classification of MsbZIP transcription factors

**Fig. 4:**
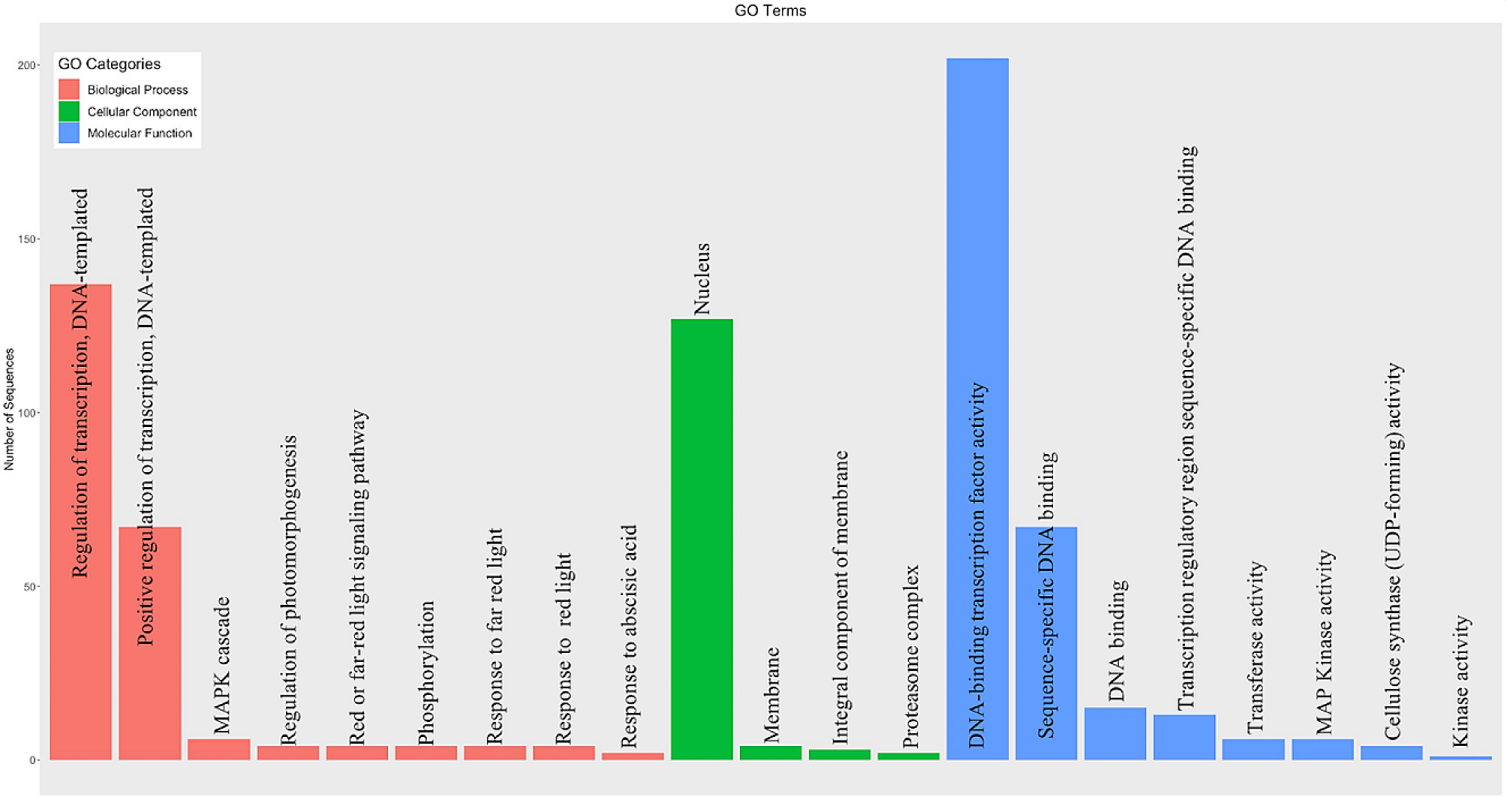
Functional annotation of bZIP proteins in alfalfa. Distribution of genes in different GO categories for Biological Process (pink), Cellular Component (green) and Molecular Function (blue). In the molecular function category, most of the genes were assigned to DNA binding transcription factor activity followed by sequence-specific DNA binding. Similarly, most of the biological process of these genes involves regulation of transcription and these genes are mostly located inside the nucleus as presented in the cellular component category

Among the 237 *MsbZIP*, 21 GO (Gene Ontology) categories were assigned to 203 of the *MsbZIPs* identified (Fig. 4). The major molecular functions of these bZIPs were DNA-binding transcription factor activity, which is consistent with their demonstrated role as transcription factors in other species. In the biological process category, most of bZIPs were assigned to the regulation of transcription category and almost all these proteins were predicted to localize to the nucleus in the cellular component category. Transcription factors provide binding sites through which they can regulate gene expression. They may act as either positive or negative regulators of downstream genes depending upon the environmental condition. The current functional classification (GO terms) of these bZIP proteins further supports their regulatory nature.

### Collinearity analysis of bZIP genes

Collinearity analysis was carried out between Alfalfa and its diploid counterpart, *Medicago truncatula*, as well as the current assembly and the latest assembly [46]. Between alfalfa and *Medicago truncatula*, 59 MsbZIPs gene pairs displayed collinearity which were distributed between four chromosomes (Chromosome 2, 4, 5 and 7) in *Medicago* (Supplementary Fig. 3). Also, the collinearity carried out between the current assembly and the new assembly of Alfalfa genome followed similar pattern which showed 77 gene pairs collinear to the new assembly in five chromosomes (Chromosome 2,4,5,7 and 8).

### Tissue-specific expression profile analysis of alfalfa bZIPs

**Fig. 5:**
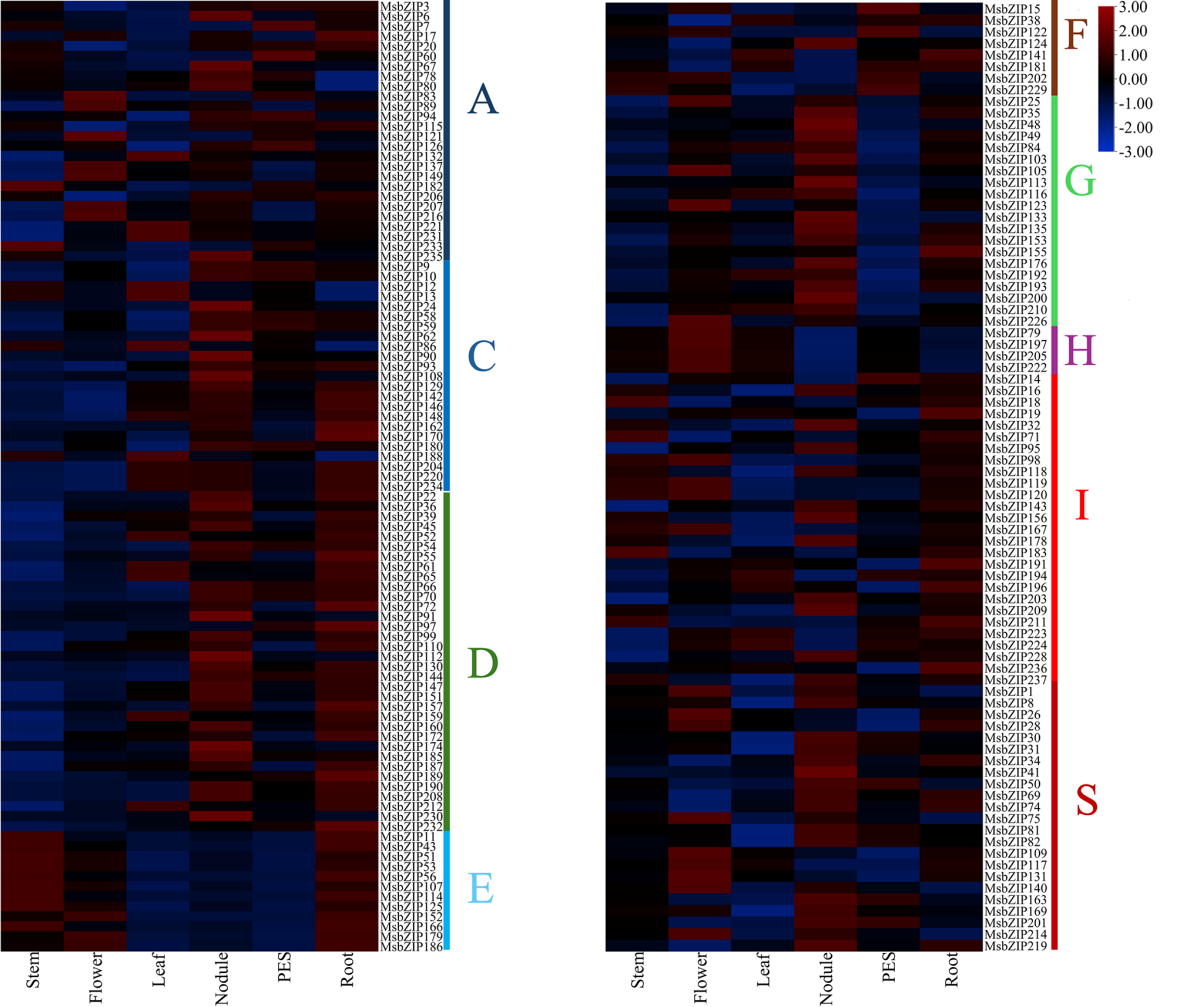
Expression profile of alfalfa bZIP genes among different tissues and organs. In the figure, PES is pre-elongated stem. Most of the genes were highly expressed in Stem, Flower, Leaf, Nodules, Pre-elongated stem (PES) and Root. The genes that were expressed in one tissue were not expressed in the other tissue indicating different genes may be required during different growth stages of alfalfa. Different groups are represented to the side of the genes by the respective group name along with the color as represented in the phylogenetic tree

After analysis of publicly available RNA-Seq data [47], we found differential expression of 177 bZIP genes. These genes were selected for having expression values in at least one of the tissues: stem, flowers, leaves, root nodules, roots, and pre-elongated stems (PES). They were then displayed in a heatmap to visualize the expression profile in different tissues and organs (Fig. 5). Differential gene expression was observed for different developmental stages. Most of the genes were highly expressed in nodules and roots. Apart from nodules and roots, genes that were upregulated in one developmental stage were downregulated in other developmental stages which can be observed in the heatmap. Even within a group, the genes were differentially expressed across all developmental stages suggesting different *bZIP* genes are required for growth and development at different stages.

### Alfalfa bZIP genes are differentially expressed in response to abiotic stresses

**Fig. 6:**
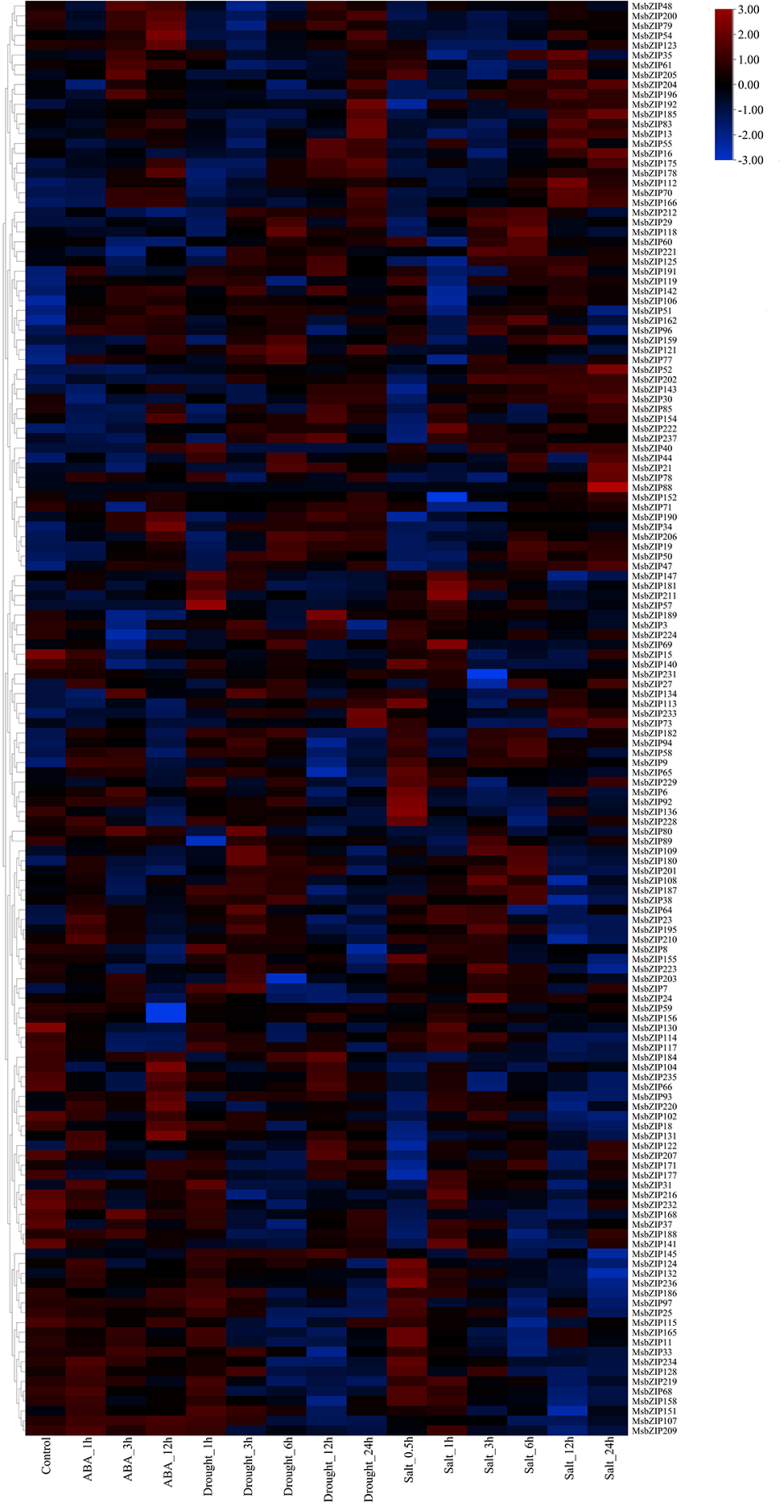
Expression profile of 146 alfalfa bZIP genes in response to ABA, drought, and salt stress. Most of the genes were highly expressed during initial treatment of salt stress from 0.5 to 3 h. For salt stress, the genes that were highly expressed during the 0.5 h of treatment were also actively expressed during 3 h of treatment. For drought stress, gene expression levels were increased as the duration of drought stress was increased from 1 h to 3 h to 6 h and more genes were expressed during 6 h of drought stress. ABA treatment showed only a few genes that were expressed as the stress duration was increased to 3 h and 12 h. Different groups are represented to the side of the genes by the respective group name along with the color as represented in the phylogenetic tree

Analysis from the publicly available RNA-seq datasets showed differential expression of 146 genes during ABA, drought, and salt stress as well as 152 bZIP proteins during cold stress at 0, 2, 6, 24, and 48 h, respectively. The expression pattern of *MsbZIP* genes during different abiotic stress conditions of cold, ABA, drought and salt showed differential expression. Across different time points of abiotic stress, the expression was different for different genes and even within a group the genes were expressed differently for different abiotic stress. Among 4 different time points of cold treatment (2 h, 6 h, 24 h and 48 h), different genes were upregulated at different time points (Supplementary Fig. 4). Even within a group, at different time points, different genes were upregulated and downregulated at different intervals of cold treatment. Like the cold treatment, abiotic stress of ABA, drought and salt treatment also showed multiple genes upregulated at different time points of stress treatment (Fig. 6). However, no genes were actively expressed during different time points of the same treatment condition among ABA, drought, and salt, which indicates different transcription factors are active during different abiotic stress as well as different time points of stress.

### RT-qPCR validation of gene expression analysis

**Fig. 7:**
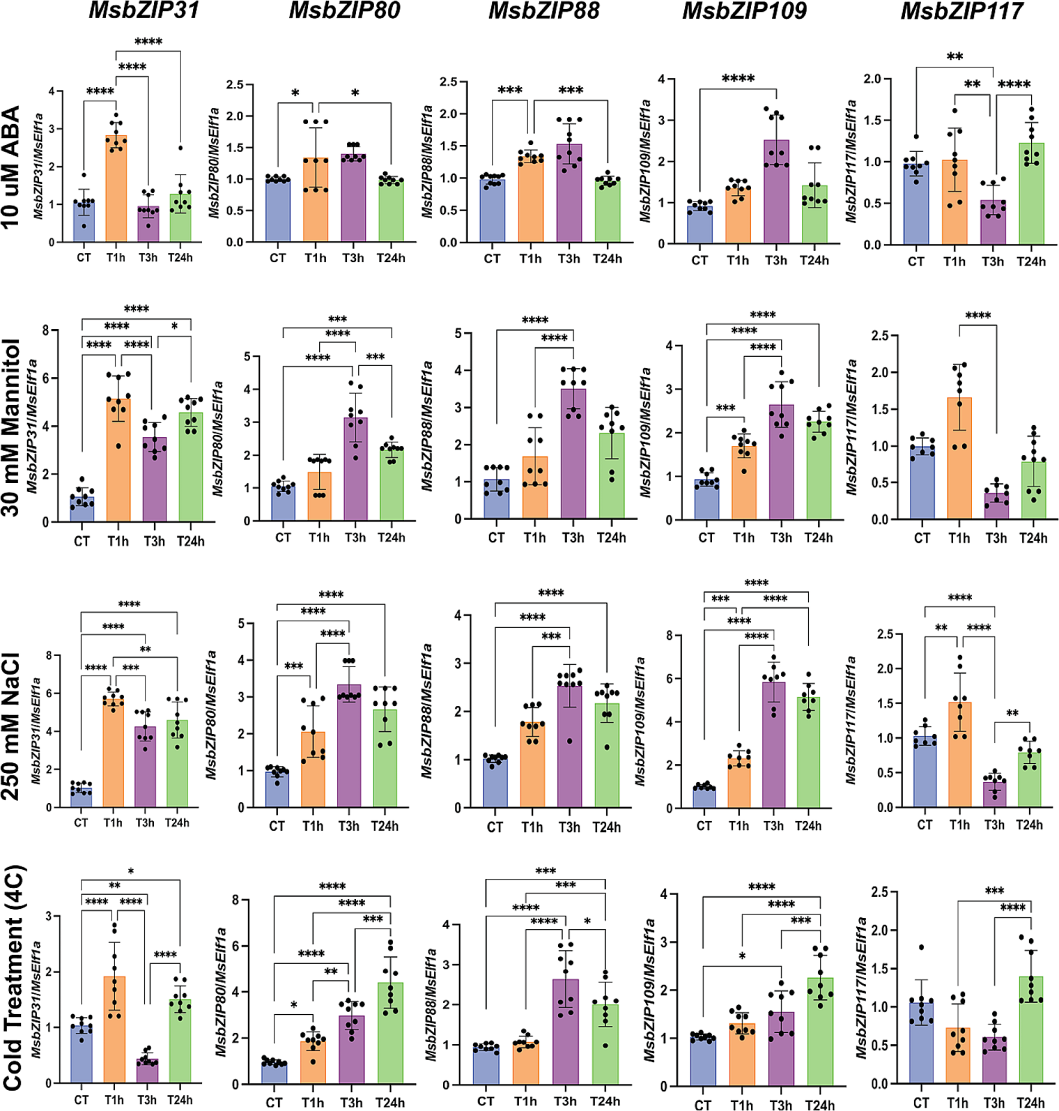
Expression analysis of five genes during abiotic stresses based on RT-qPCR. Columns represents individual genes, while rows represent four stress conditions (cold, drought, salt, and ABA). The different treatment time point of 0-hour (CT), 1 h (T1h), 3 h (T3h) and 24 h (T24h) is presented in x-axis, while y-axis presents the expression value. A line is used to show the significant expression at different level of significance (0.05, 0.01, 0.001)

For RNA-Seq result verification, five differentially expressed genes, two from group A (*MsbZIP80* and *MsbZIP88*) and three from group S (*MsbZIP31*, *MsbZIP 109* and *MsbZIP117*) were selected for RT-qPCR analysis. The expression pattern for most of the genes were consistent with the RNA-Seq analysis (Fig. 7). In addition, the genes also showed high group specificity, as genes from Group A (*MsbZIP80* and *MsbZIP88*) and Group S (*MsbZIP31*, *MsbZIP 109* and *MsbZIP117*) were consistent in expression to their specific groups. Significant upregulation of all five genes were found at the 1-hour timepoint for salt stress. *MsbZIP31* and *MsbZIP117* expression then decreased at the 3 and 24-hour timepoints compared to the 1-hour timepoint, whereas the genes from the A- *MsbZIP80* and *MsbZIP88* (A Group) were the most upregulated at the 3-hour timepoint.

Similarly, for drought treatments, all the genes except *MsbZIP117*, were highly upregulated upon 1-hour of exposure of mannitol and remained upregulated relative to the control. However, *MsbZIP117* was downregulated at 3-hours and then returned to normal expression levels at 24 h. Similar to drought treatment and as expected, the same trend of upregulation and downregulation for all five genes continued during their treatment of ABA across the timepoints.

For the cold treatments, *MsbZIP80* and *MsbZIP109* were continuously upregulated when exposed to 4 °C at all the timepoints (from 1-h to 24-h). For the remaining three genes (*MsbZIP31, MsbZIP88* and *MsbZIP117*), they followed completely different trend across the samples and treatments. While *MsbZIP31* was significantly upregulated at 1-hour of exposure of cold, *MsbZIP117* was downregulated and *MsbZIP88* did not change. However, *MsbZIP88* was significantly upregulated from 1-hour to 3-hour treatment while the two genes downregulated at the same treatment. *MsbZIP31* and *MsbZIP117* were then upregulated from 3-hour to 24-hour of treatment. *MsbZIP88* showed slight but significant upregulation in response to cold at the 3-hour timepoint. The level of significance during upregulation and downregulation of genes is presented in Fig. 7.

**Fig. 8:**
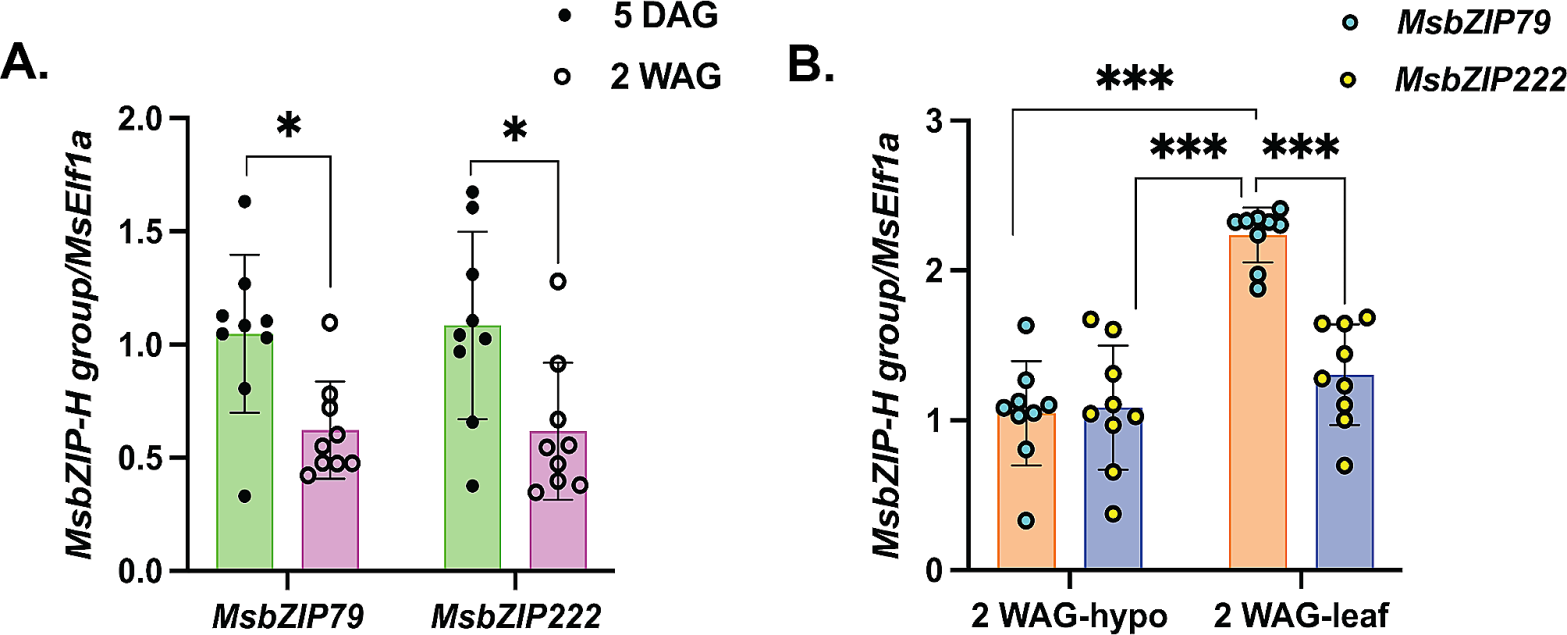
Expression analysis of genes for developmental stages. (**A**) represents tissues extracted from 5 days after germination (DAG) and 2 weeks after germination (WAG), while (**B**) represents tissues extracted from 2 weeks seedlings from hypocotyl and leaf. A horizontal line showing significant expression at different level of significance (0.05, 0.01, 0.001) is also presented

To validate the tissue specific expression of bZIPs, genes from Group H (*MsbZIP79* and *MsbZIP222*) were selected. In comparison to the 5-day old hypocotyl, 2-week-old hypocotyl of both *MsbZIP79* and *MsbZIP222* were significantly downregulated (*p* < 0.01) (Fig. 8). However, for both the genes, they were upregulated in leaf tissue in comparison to the hypocotyl tissues extracted from 2-week-old seedlings. For *MsbZIP79*, the upregulation was highly significant (*p* < 0.001); however, it was not the same for *MsbZIP222*. *MsbZIP79* was significantly upregulated in 2-week-old leaves compared to 2-week-old hypocotyls. The expression pattern of these genes was also in consistent with the RNA-Seq analysis.

### Cis-regulatory elements in bZIP gene promoter

The expression pattern of stress responsive-genes are often controlled by cis-regulatory elements. These elements are typically located 5’ upstream of the gene coding sequences. These elements provide a binding site for the transcription factors to switch on or off the gene based on the environmental condition. In this study, we analyzed 135 stress-responsive bZIP promoters, we identified 875 cis-regulatory elements distributed along these 135 bZIP promoter. The detailed distribution of these cis-elements along the bZIP promoters was performed (Supplementary Fig. 5). We focused on cis-elements implicated in abiotic stress responses and found an abundance of the following cis-regulatory elements: abscisic acid responsive element (ABRE), methyl jasmonate responsive motif (CGTCA-motif), light inducible G-box motif, low-temperature responsive (LTR), drought responsive (MBS binding site) and defense and stress responsive (TC-rich repeats). Among the 875 cis-elements, light inducing G-box motif was the highest with 274 followed by abscisic stress responsive element (ABRE) with 234 while low temperature responsive (LTR) with 50 was the lowest.

## Discussion

In the present study, we identified 237 bZIP sequences from tetraploid alfalfa that contained both a highly conserved basic region and the heptad repeat leucine zipper region, suggesting they are functional bZIPs. As predicted, the number of bZIP in tetraploid alfalfa (237) is more than double to that of diploid model legume *M. truncatula* [43]. Not surprisingly, the number of bZIP genes varied amongst plant species with *(A) thaliana* (78), *L. japonicus* [42], *M. truncatul*a [43] and *O. sativa* (89) [6, 8, 10, 41, 48, 49]. Similarly, the allotetraploid *(B) napus* genome contained 247 bZIP genes, which is roughly double that of the number found in the related diploid *A. thaliana*. There has been previous study of bZIP genes in Alfalfa [50], where 57 MsbZIPs were identified. However, given the tetraploid genome of alfalfa and compared with its diploid counterpart, the number of bZIPs in the current study looks comprehensive to the previous study.

Based on phylogenetic analysis and previous analyses from *A. thaliana*, *M. truncatula*, *L. japonicus* and *O. sativa*, we classified the alfalfa bZIP genes into 10 groups (A, C, D, E, F, G, H, I, M, and S). The most recent classification of bZIPs from *A. thaliana* [8] sorted *AtbZIPs* into 13 groups. Notably, groups B, J and K are missing in our analysis of alfalfa. In *A.thaliana* there are three members of group B (bZIP17, bZIP28, and bZIP49) and one group K member (bZIP60), which are implicated in endoplasmic reticulum stress responses [51], but both these groups are missing in alfalfa which begs the question of which groups perform this function in alfalfa. Group J in *A. thaliana* is made up of a single copy gene, bZIP62, which is related to Group G bZIP GBF1– a negative regulator of blue-light responsive hypocotyl growth that acts antagonistically to HY5 and HYH, two group H bZIPs important in photomorphogenic growth [18, 52]. Another remarkable difference between groups is the group M bZIP72, which is single copy in *A. thaliana* but contains 13 members in alfalfa. It will be interesting to determine the role M group bZIPs play in alfalfa and it is intriguing to postulate why this group has increased in number.

It is well established that bZIP transcription factors have a myriad of roles in plant development such as seed maturation and germination [18], floral induction and development [21, 24]. Not surprisingly, tissue-specific expression of 177 bZIP genes in nodules, flowers, roots, leaves, and stems was found in alfalfa as well (Fig. 5). Interestingly, group E members were most specifically expressed in stems, roots, and flowers, whereas several group F members were expressed in pre-elongated stems. In *A. thaliana* the group E member bZIP34 has been linked to pollen germination and pollen tube growth [23]. In contrast, group F members regulate zinc (Zn) transporters and salt stress responses [53, 54]. Group C and S bZIPs are known to heterodimerize in the so-called C/S1 bZIP network involved in nutrient and energy metabolism [28, 53]. Likewise, group C and S bZIPs are co-expressed in some tissues such as roots and nodules in alfalfa.

In addition to regulating development, bZIPs play a wide array of roles in biotic and abiotic stress responses in different crop species [54]. identified the *OsABI5* bZIP TF that was involved in rice fertility and stress tolerance [7]. related bZIP genes in rice to drought tolerance through genomic survey and gene expression analysis. Similarly, a root-specific bZIP transcription factor was isolated in tepary beans and found to be responsive to water-stress conditions [55] [12]. isolated three bZIP genes (*GmbZIP44, GmbZIP62, GmbZIP78*) and found a negative regulator of ABA and tolerance to salt and freezing stress by overexpression in *A. thaliana*. As several studies have shown the role of bZIP transcription factors in the response to plant stress [36], further added to it by cloning a bZIP gene and measuring physiological changes mediated by it in alfalfa under different stress conditions. Additionally, the over-expressed cloned Alfalfa *bZIP* genes in tobacco plants resulted in transgenic tobacco plants conveying salt and drought tolerance. These results indicate that the over-expression of certain *bZIP* genes increases the tolerance of plants to different abiotic stresses.

Furthermore, RT-qPCR analysis was carried out to corroborate the expression trends from RNA-Seq analyses. The genes selected for abiotic stress were from Group A (*MsbZIP80,MsbZIP88*) and Group S (*MsbZIP31, MsbZIP109,MsbZIP117*), while Group H (*MsbZIP79,MsbZIP222*) genes were selected for expression during developmental stages. In *A. thaliana*, Group A genes encode abscisic acid-responsive element binding factors (ABF1) that act at the core of the ABA signaling pathway [56]. During water deficit conditions like cold, salt and drought, these factors are induced for the adaptive response to overcome water deficit conditions [56]. Similarly, expression analysis of *Medicago truncatula* revealed *bZIP* genes that were responsive to drought and salt stress conditions were concentrated in Group A and S [49]. Furthermore, *bZIPs* from these groups were found to be involved in sugar signaling process [57], resulting in physiological and developmental changes, which integrates with other signaling pathways in plants for stress response [57]. Similar to these studies, we also found the *MsbZIPs* from Group A and Group S were highly induced with significant expression during differential treatment of salt, cold, drought and ABA.

In *A. thaliana*, group H bZIPs contain elongated hypocotyl (HY5) and the HY5 homologue (HYH), which have been found to play important roles in developmental process [8]. HY5 regulates developmental process through cell elongation, cell proliferation, chloroplast development, pigment accumulation and nutrient assimilation [58]. These genes inhibit hypocotyl elongation in light and promote plant growth by inducing nutrient uptake and through expression of enzymes associated with nitrogen, sulfur and copper required for overall growth [59]. The findings of the current study revealed that the *bZIPs* in Group H (*MSbZIP79,MsbZIP222*) are significantly downregulated in 2 weeks old hypocotyl tissue in comparison to 5 day-hypocotyl tissue. However, these genes were more highly expressed in 2-week-old leaf samples, which further establishes their role in the developmental processes in leaves as has been proposed in *A. thaliana.*

## Conclusion

Here we report the comprehensive *in silico* analysis of the bZIP transcription factor family in alfalfa (*M. sativa*). We identified 237 *bZIP* genes and named them *MsbZIP1* to *MsbZIP237*. Phylogenetic analysis of these bZIP genes using *A. thaliana* as a reference divided the sequences into 10 groups, with B, J, and K missing in alfalfa. The physicochemical analysis and motif analysis showed high specificity within each group. The expression profile of bZIPs suggests bZIPs are expressed in a tissue-specific manner. Finally, the expression profiles of bZIP genes during different abiotic stress conditions (cold, ABA, drought, and salt) showed the specific response of a few bZIP at specific time points during the stress response making them good candidates for stress-responsive transcription factors and further functional characterization. Taken together, this work provides a framework for the future study of bZIPs in alfalfa and presents candidate bZIPs involved in stress-response signaling.

## Materials and methods

### Identification of bZIP transcription factor gene family in alfalfa

For comprehensive identification and analysis of the bZIP transcription factor (TF) gene family in alfalfa, the sequences of bZIP transcription factors from model and known species were downloaded from the Plant Transcription factor database (http://planttfdb.cbi.pku.edu.cn/), which included 127 sequences from *Arabidopsis thaliana*, 93 from *Lotus japonicus*, 124 from *Medicago truncatula* and 140 from *Oryza sativa*. The number of bZIPs used were more than that is mentioned in Table 1 as it included spliced variants as well. A local protein database was created using Basic Local Alignment Search Tool [60] with protein sequences from chromosome level assembly of alfalfa [40]. A BLASTp search was conducted in the local database created using the protein sequences from alfalfa, taking the bZIP sequences from model organisms as a query with an E-value cut-off of 1E-05 (0.00001). The bZIP sequences obtained from the search were further confirmed based on the presence of the bZIP domain (N-x [7]-R/K-x [9]-L-x [6]-L-x [6]-L) using the Pfam web program (https://pfam.xfam.org/) with an E-value of 1.0. Further, the bZIP domain was used to search against the database of the identified bZIP sequences using the Prosite program of the ExPASy bioinformatics resource (http://protsite.expasy.org). The identified sequences with intact bZIP domains were predicted to be bonafide bZIP sequences.

### Evolutionary analysis, protein properties and detection of conserved motifs in the bZIPs

To analyze the sequence features of bZIP transcription factors, multiple sequence alignment of 237 bZIP proteins were performed using multiple sequence comparison by log-expectation (MUSCLE) [61] command using default parameters. The output of the multiple sequence alignment was visualized using Unipro UGENE v.33 [62]. For evolutionary analysis, 549 sequences were used which included sequences from *M. sativa* (237), *A. thaliana* (78), *L. japonicus* [42], *M. truncatula* [43] and *O. sativa* (89). Multiple sequence alignment was carried out by CLUSTALW with default parameters. Subsequently, the phylogenetic tree was constructed by the Neighbor Joining method using 1000 bootstraps replicates. Phylogenetic analyses were conducted using MEGA version X [63].

Other physical properties of the identified sequences like the molecular weight and theoretical isoelectric point (pI) were determined using Compute pI/Mw tools (http://web.expasy.org/compute_pi/) of ExPASy bioinformatics resource. The MEME [45] program was used to identify the conserved motifs within the full-length Alfalfa. The parameters used were maximum number of motifs to be 25, distribution of motifs = any number of repetitions, optimum motif width = 6 to 50 residues.

### In silico functional analysis of bZIP genes

For predicting the MsbZIP protein function (gene ontology) GO annotation was performed using the web-accessible Blast2GO v4.1 annotation system (https://www.blast2go.com/) [64]. Briefly, the MsbZIP protein sequences were used to search for similar sequences against the NCBI non-redundant (Nr) database using the Blast tool in the Blast2GO software, with an E-value of 10 − 3 (1e-03). Next, mapping and annotation were performed on Blast2GO using default parameters. Finally, functional classification was also performed by Blast2GO.

### Collinearity analysis in MsbZIP genes

The collinearity relationship of MsbZIP genes with gene pairs from its closest relative, *Medicago truncatula* was carried out using TBtools with Multiple Collinearity Scan toolkit (MCScanX) [65, 66]. In addition, the collinearity between the current assembly and new assembly [46] of alfalfa was also carried out. Finally, the results from the collinearity analysis were visualized using TBtools.

### Expression analysis during plant development

The raw RNA sequence data was downloaded from the National Center for Biotechnology Information (NCBI) Sequence Read Archive (SRA), SRP055547 [47]. The data was generated from six tissues at different growth stages of Alfalfa namely, root, nodule, elonged stem, pre-elonged stem, leaf, and flower. The tissue sample for RNA-Seq was collected at the respective stage of alfalfa plants. Fastqc version 0.11.7 was used for quality check of the raw sequences. The reads passing the minimum Phred quality score of 30 were selected. The RNA-Seq analysis was carried out following the method described by [67], in which the filtered reads were aligned with the reference genome using HISAT2 version 2.1.0 [68] and sorted by Samtools ver 1.9 [69]. Transcript assembly and quantification was carried out using Stringtie version 2.1.1 [70]. A python script was used to extract read count information directly from the files generated from Stringtie and edgeR package [71] in R was used for differential gene expression analysis. TBtools version 1.0692 [66] was used to generate heatmaps for the differentially expressed genes.

### Transcriptome analysis of bZIP genes in response to abiotic stresses

The raw RNA sequence data from previous studies were downloaded from the National Center for Biotechnological Information (NCBI) Sequence Read Archive (SRA). The transcriptome data consist of cold treatment (SRR7091780-SRR7091794 [42]), and ABA, drought, and salt treatments (SRR7160313-SRR7160357) [72, 73]. All these samples were collected from 12 days old alfalfa seedlings for RNA-Seq. Fastqc version 0.11.7 was used for quality check of the raw sequences. The reads passing the minimum Phred quality score of 40 were selected. The RNA-Seq analysis was carried out following the method described by [67], in which the filtered reads were aligned with the reference genome using HISAT2 ver2.1.0 and sorted by Samtools ver1.9. Transcript assembly and quantification was carried out using Stringtie version 2.1.1. A python script was used to extract read count information directly from the files generated from Stringtie and edgeR package in R was used for differential gene expression analysis. TBtools version 1.0692 was used to generate heatmaps for the differentially expressed genes.

### Analysis of cis-regulatory elements

For this analysis, the bZIP genes with changed expression during abiotic stress were visualized using Integrated Genome Browser 9.1.4 [74] to locate the promoter sequences. Samtools (ver. 1.9) was used to extract the 2000 bp sequence from the promoter of these changed bZIP genes to investigate the potential cis-regulatory elements by querying them through the PlantCARE database (http://bioinformatics.psb.ugent.be/webtools/plantcare/html/). In total six cis-regulatory elements responsive to stress were analyzed. These elements included abscisic acid responsive (ABRE), methyl jasmonate responsive (CGTCA-motif), light inducible G-box motif, low-temperature responsive (LTR), drought responsive (MBS binding site) and defense and stress responsive (TC-rich repeats).

### Plant materials and RNA isolation

An alfalfa (*Medicago sativa*) variety Vernal was collected from Washington State University, Pullman, WA. Seeds were sterilized using 70% ethanol inside the laminar hood and kept in 4 °C dark for 3 days. After that, seeds were transplanted in half strength Murashige and Skoog’s medium (PhtyoTech Labs, KS) containing 3% sucrose and 0.7% agar inside a growth chamber with light cycle of 16:8-h (light/dark) and temperature of 22 °C. We simulated abiotic stress with abscisic acid (ABA, 10 µM), NaCl (250 mM), mannitol (300 mM) and 4 °C cold stress treatment at 2 weeks after germination (WAG). Leaf tissue samples were collected at the following time points: 0 (control, CT), 1, 3, and 24 h for selected *MsbZIP* gene expression analysis. For selected *MsbZIP* H-group gene expression analysis, we collected hypocotyl samples at 5 days after germination (DAG) and 2 WAG. The collected samples were quickly placed in liquid nitrogen and stored in a − 80 °C freezer for subsequent RNA extraction.

Total RNA was extracted using the Spectrum^™^ plant total RNA isolation kit (Sigma Aldrich, USA) and treated with DNaseI to eliminate genomic DNA contamination. Total RNA yield (ng/µL) and purity (260:280 wavelength ratios) was measured by using Nanodrop (Eppendorf, USA) instrument. 2 µg RNA from each of the samples were used for the synthesis of single stranded cDNA. We used Bio-Rad iScript™ (Hercules, CA, USA) for cDNA synthesis.

### Reverse transcription quantitative real-time PCR analysis

Reverse Transcription Quantitative real-time PCR was performed with volumes of 10 µL per well with Bio-Rad™ SYBR green Supermix (Hercules, CA, USA). The amount of cDNA was normalized to the level of *Medicago sativa* housekeeping gene *elongation factor 1α* (*MsElf1α*) used as an internal control. The amplification was conducted in Biorad 96 Real-Time PCR System. A standard thermal profile for SYBR green mix was as followed: cDNA synthesis at 37 °C for 15 min and enzyme inactivation at 85 °C for 5 s. qPCR conditions were: initial denaturation 96 °C for 30s, denaturation 96 °C for 5 s, annealing and extension 62 °C for 30s. Transcripts expression levels were calculated with the 2 − ΔΔCt method, as previously mentioned in [75]. Three biological and three technical replicates were used for gene expression analysis. Primers used for this analysis are mentioned in Supplementary Table 3.

### RT-qPCR data analysis

One-way ANOVA followed by multiple comparisons test was performed [43] using GraphPad Prism version 8.0 Statistics for all selected *MsbZIP* candidate gene expression analysis used in RT-qPCR validation.

### Electronic supplementary material

Below is the link to the electronic supplementary material.

Supplementary Material 1

Supplementary Material 2

Supplementary Material 3

Supplementary Material 4

Supplementary Material 5

Supplementary Material 6

Supplementary Material 7

Supplementary Material 8

Supplementary Material 9

Supplementary Material 10

## Data Availability

The datasets generated and analyzed during the current study are available in the, Plant Transcription Factor database (http://planttfdb.gao-lab.org/family.php?fam=bZIP), Zeng, Yan (2020): genome fasta sequence and annotation files. figshare. Dataset., 10.6084/m9.figshare.12327602.v3. Raw reads for RNA-seq are available with Sequence Read Archive SRP055547, SRR7091780 to SRR7091794, and SRR7160313 to SRR7160357.

## Abbreviations

ABA: Abscisic acid
BLAST: Basic local alignment search tool
bZIP: Basic-leucine zipper
DNA: Deoxyribonucleic acid
GO: Gene ontology
Mb: Mega base
MEGA: Molecular evolutionary genetics analysis
MEME: Multiple Em for Motif Elicitation
MUSCLE: Multiple Sequence Comparison by Log-Expectation
NASS: National Agricultural Statistics Service
NCBI: National center for biotechnology information
PES: Pre-elongated stem
RNA-Seq: Ribonucleic acid sequencing
RT-qPCR: Reverse Transcription Quantitative Real-time PCR
SAR: Systemic acquired resistance
SRA: Sequence read archive
TFs: Transcription Factors
TGA: TGACG-Binding
USDA: United States Department of Agriculture
Zn: Zinc
