# Supplementary figures and images for: In silico analysis identified bZIP transcription factors genes responsive to abiotic stress in Alfalfa (Medicago sativa L.)
Source: BMC Genomics. 2024 May 21;25:497. doi: 10.1186/s12864-024-10277-3 (PMC11106943; doi:10.1186/s12864-024-10277-3)

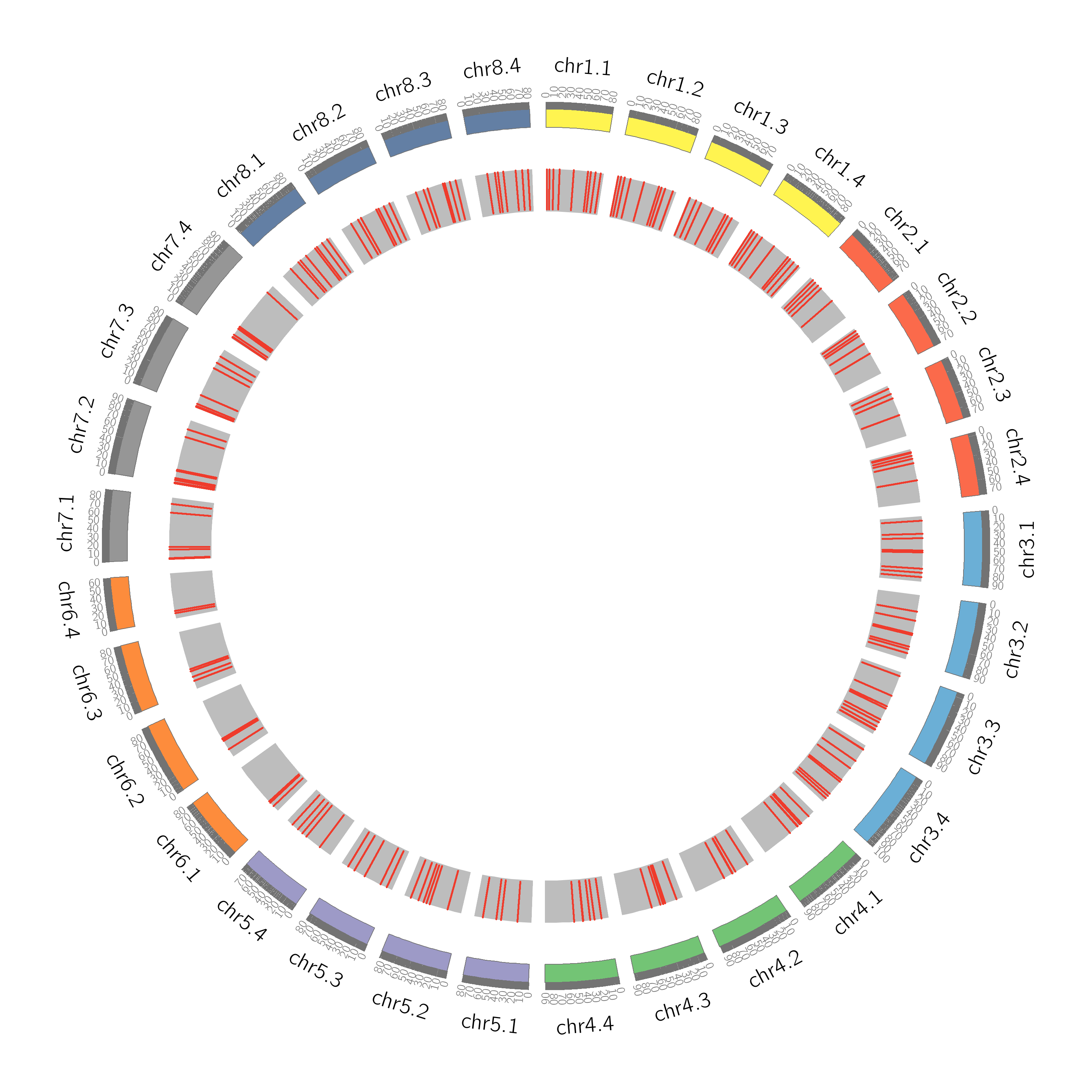

Supplement: Supplementary file 4 — Supplementary Material 4 [file 12864_2024_10277_MOESM4_ESM.jpg]

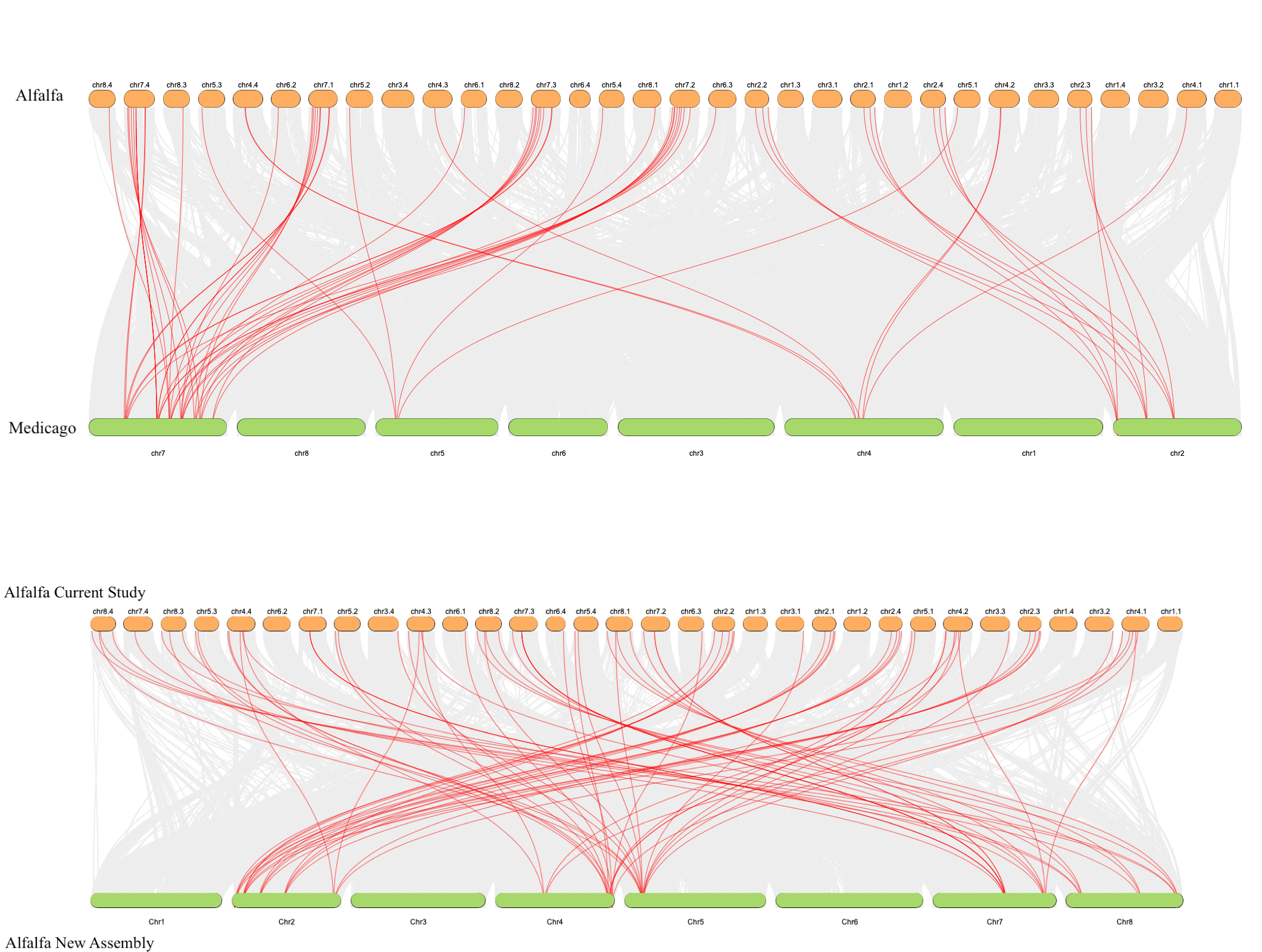

Supplement: Supplementary file 5 — Supplementary Material 5 [file 12864_2024_10277_MOESM5_ESM.jpg]

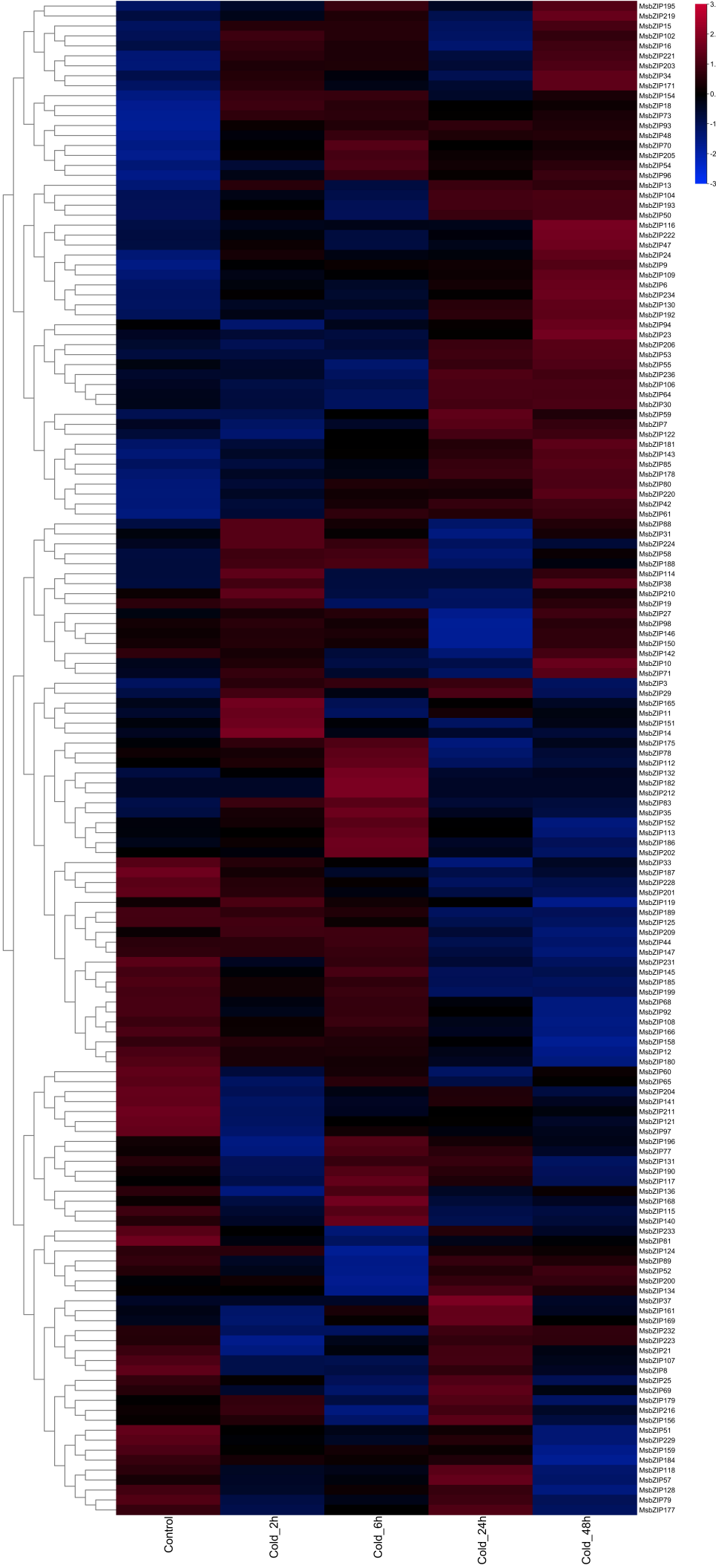

Supplement: Supplementary file 6 — Supplementary Material 6 [file 12864_2024_10277_MOESM6_ESM.pdf]

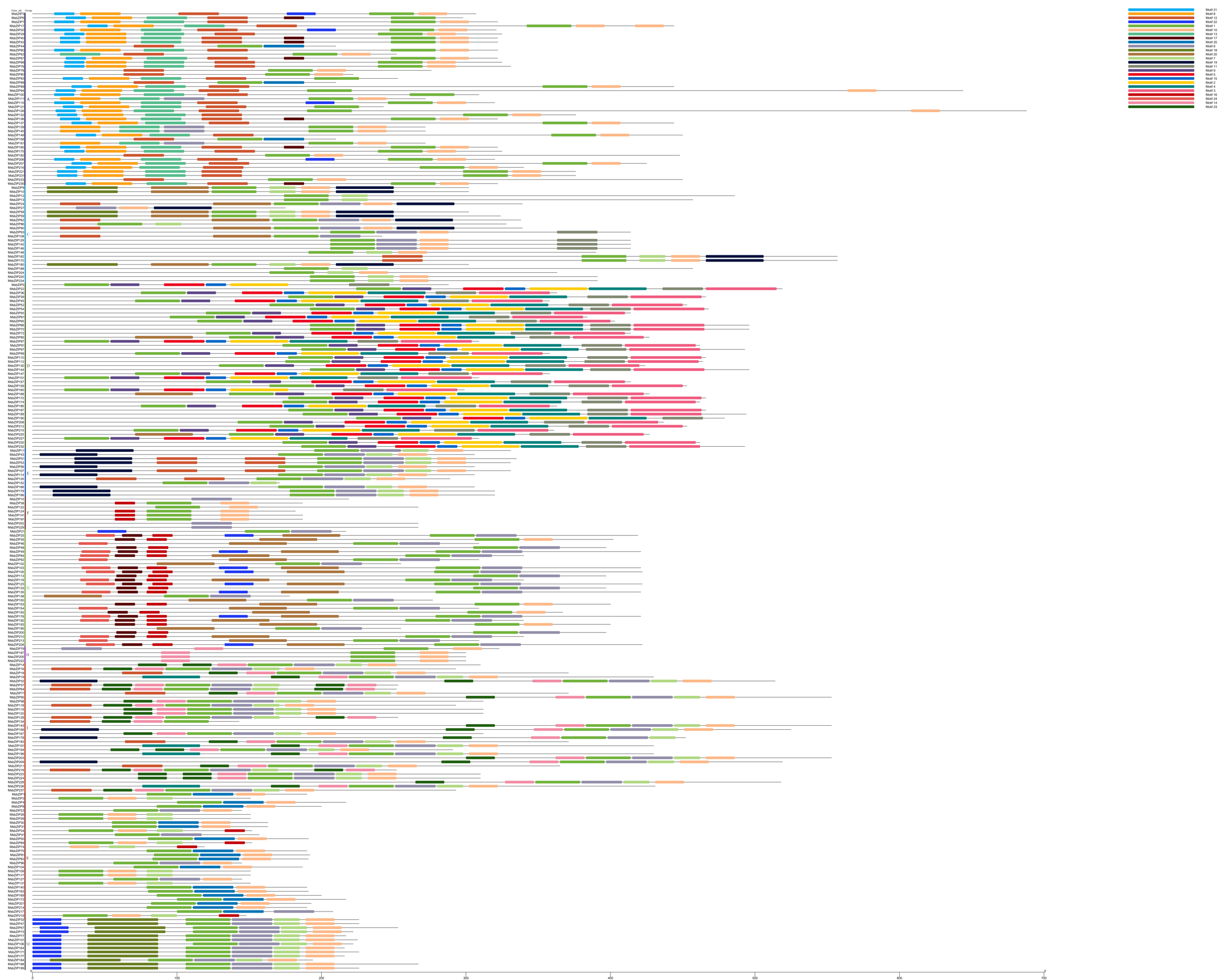

Supplement: Supplementary file 8 — Supplementary Material 8 [file 12864_2024_10277_MOESM8_ESM.pdf]
