# Supplementary material for: In silico analysis identified bZIP transcription factors genes responsive to abiotic stress in Alfalfa (Medicago sativa L.)
Source: BMC Genomics. 2024 May 21;25:497. doi: 10.1186/s12864-024-10277-3 (PMC11106943; doi:10.1186/s12864-024-10277-3)
Supplement: Supplementary file 7 — Supplementary Material 7 [file 12864_2024_10277_MOESM7_ESM.pdf]

ids	ABRE	CGTCA Motif	G-Box	LTR	MBS	TC-rich repeats
MsbZIP3	0	0	0	0	1	0
MsbZIP6	6	0	6	0	0	1
MsbZIP7	8	0	10	0	1	0
MsbZIP29	1	1	3	1	2	0
MsbZIP44	1	2	0	0	0	0
MsbZIP60	1	1	1	1	2	1
MsbZIP68	2	1	3	1	3	0
MsbZIP78	1	2	0	1	0	0
MsbZIP80	3	0	6	1	0	0
MsbZIP83	2	0	2	2	0	1
MsbZIP88	1	2	0	0	0	0
MsbZIP89	2	1	3	0	0	0
MsbZIP94	2	0	2	2	0	1
MsbZIP115	0	1	0	0	1	0
MsbZIP121	2	0	2	2	0	1
MsbZIP132	1	1	2	0	2	0
MsbZIP136	3	0	2	0	1	1
MsbZIP145	0	1	0	0	1	0
MsbZIP158	1	2	0	0	0	0
MsbZIP165	0	0	1	0	2	1
MsbZIP175	1	1	3	1	1	0
MsbZIP182	3	0	6	1	0	0
MsbZIP206	1	0	1	0	1	0
MsbZIP216	2	1	3	0	0	0
MsbZIP221	1	1	2	0	2	0
MsbZIP231	1	0	2	0	2	1
MsbZIP233	1	0	3	1	0	0
MsbZIP9	5	2	3	0	0	1
MsbZIP13	2	2	2	0	0	0
MsbZIP24	0	0	0	1	0	0
MsbZIP27	2	1	4	1	1	0
MsbZIP58	5	2	3	0	0	1
MsbZIP59	5	2	3	0	0	1
MsbZIP93	0	2	0	0	3	0
MsbZIP108	1	0	1	2	0	0
MsbZIP142	1	1	0	2	0	0
MsbZIP180	5	2	3	0	0	1
MsbZIP188	3	6	3	1	0	0
MsbZIP204	1	0	2	0	2	0
MsbZIP220	1	0	1	0	2	0
MsbZIP234	1	0	1	0	2	0
MsbZIP52	5	3	8	1	0	0
MsbZIP54	2	1	2	0	1	0
MsbZIP55	2	1	2	0	0	2
MsbZIP61	1	2	1	3	1	0
MsbZIP65	2	1	2	0	0	2
MsbZIP70	2	1	2	0	0	0
MsbZIP85	0	1	1	1	0	1
MsbZIP97	1	0	1	0	0	1
MsbZIP112	0	1	0	0	0	0
MsbZIP130	4	1	3	0	1	2
MsbZIP147	0	2	0	0	0	1
MsbZIP151	0	2	1	0	0	0
MsbZIP159	5	2	8	1	0	0
MsbZIP168	0	1	1	1	0	1
MsbZIP185	3	1	4	0	0	1
MsbZIP187	1	0	1	0	2	1
MsbZIP189	1	0	1	0	0	1
MsbZIP190	2	0	3	1	0	0
MsbZIP212	0	3	0	0	2	1
MsbZIP232	1	0	1	0	0	1
MsbZIP11	4	0	4	0	1	0
MsbZIP51	2	0	2	0	1	1
MsbZIP107	2	0	2	0	1	1
MsbZIP114	4	0	4	0	1	0
MsbZIP125	4	1	4	0	1	0
MsbZIP152	2	1	3	1	0	0
MsbZIP166	4	0	4	0	1	0
MsbZIP186	4	1	4	1	0	0

ids	ABRE	CGTCA Motif	G-Box	LTR	MBS	TC-rich repeats
MsbZIP15	0	0	0	1	0	1
MsbZIP38	0	0	1	0	3	0
MsbZIP122	0	1	0	0	2	0
MsbZIP124	0	0	0	0	2	1
MsbZIP141	0	0	1	0	2	1
MsbZIP181	1	0	2	0	4	0
MsbZIP202	1	2	1	1	2	1
MsbZIP229	0	2	0	0	2	0
MsbZIP21	4	0	4	0	1	0
MsbZIP25	12	1	11	1	1	1
MsbZIP35	2	5	3	0	0	0
MsbZIP48	2	1	2	0	0	1
MsbZIP92	1	2	1	0	0	1
MsbZIP102	1	0	0	0	1	1
MsbZIP113	2	1	2	0	0	1
MsbZIP154	4	3	4	0	0	3
MsbZIP192	3	2	2	0	0	1
MsbZIP195	3	2	5	0	2	0
MsbZIP200	2	1	2	0	0	1
MsbZIP210	1	0	3	1	1	0
MsbZIP79	0	1	1	0	1	0
MsbZIP205	0	1	1	0	1	1
MsbZIP222	0	1	1	0	1	1
MsbZIP16	2	3	1	0	1	1
MsbZIP18	4	1	5	1	1	0
MsbZIP19	3	0	3	1	0	0
MsbZIP37	2	3	2	0	2	0
MsbZIP64	2	2	2	1	1	1
MsbZIP71	4	1	5	0	1	0
MsbZIP118	0	1	0	1	4	0
MsbZIP119	0	1	0	0	1	1
MsbZIP128	2	2	2	1	1	1
MsbZIP134	0	0	0	1	0	1
MsbZIP143	0	4	0	0	0	0
MsbZIP156	1	1	1	0	1	1
MsbZIP178	5	2	8	1	0	0
MsbZIP196	0	0	1	0	0	0
MsbZIP203	2	3	3	0	0	0
MsbZIP209	0	1	2	0	1	1
MsbZIP211	1	0	1	0	1	1
MsbZIP224	0	1	0	1	0	1
MsbZIP228	0	4	0	0	0	0
MsbZIP236	2	0	2	2	0	0
MsbZIP8	0	1	1	0	1	0
MsbZIP23	0	0	0	0	1	1
MsbZIP30	0	0	0	1	0	0
MsbZIP31	4	2	2	0	2	0
MsbZIP50	3	3	3	1	1	1
MsbZIP69	1	1	1	0	0	0
MsbZIP96	2	1	2	0	0	0
MsbZIP104	0	1	0	0	0	0
MsbZIP109	0	1	0	0	1	0
MsbZIP117	2	1	2	0	1	1
MsbZIP131	2	1	2	0	2	0
MsbZIP140	2	3	1	0	0	2
MsbZIP201	2	1	5	0	0	1
MsbZIP219	1	0	1	0	0	1
MsbZIP33	1	0	1	2	0	1
MsbZIP47	1	0	1	0	0	2
MsbZIP57	2	2	3	0	2	0
MsbZIP73	0	1	0	0	0	1
MsbZIP77	0	1	0	0	0	1
MsbZIP106	1	1	1	0	1	1
MsbZIP171	2	0	5	0	1	0
MsbZIP177	2	4	2	0	0	1
MsbZIP184	0	1	0	1	0	2

Group

A
C
D
E
F
G
H
I
S
M
