# Supplementary material for: In silico analysis identified bZIP transcription factors genes responsive to abiotic stress in Alfalfa (Medicago sativa L.)
Source: BMC Genomics. 2024 May 21;25:497. doi: 10.1186/s12864-024-10277-3 (PMC11106943; doi:10.1186/s12864-024-10277-3)
Supplement: Supplementary file 9 — Supplementary Material 9 [file 12864_2024_10277_MOESM9_ESM.docx]

**Supplementary Table 1 Summary of the bZIP transcription factor genes in Alfalfa.**

**Supplementary Table 2 Meme motif sequences in predicted bZIP protein**

| Motif | E-value | Width | Top scoring sequence |
| --- | --- | --- | --- |
| 1 | 0.00E+00 | 32 | TVERRQKRMIKNRESAARSRARKQAYTQELEI |
| 2 | 0.00E+00 | 41 | HYDEIFRLKGVAAKADVFHLLSGMWKTPAERCFLWLGGFRS |
| 3 | 0.00E+00 | 50 | FIRQADNLRQQTLQQMHRILTTRQSARALLAIHDYFSRLRALSSLWLARP |
| 4 | 0.00E+00 | 41 | SELLKLLVSHLEPLTEQQLMGIYNLQQSSQQAEDALSQGMD |
| 5 | 0.00E+00 | 29 | NGAMAFDAEYARWLEEHNRQTNELRAAIN |
| 6 | 0.00E+00 | 29 | KVQTLQTEATTLSAQLTLYQRDTTGLSTE |
| 7 | 0.00E+00 | 19 | NTELKLRLQAMEQQAHLRD |
| 8 | 0.00E+00 | 29 | MGGSGKDFGSMNMDELLKNIWSAEEVQTM |
| 9 | 0.00E+00 | 21 | SRLKLTQLEQELQRARQQGVF |
| 10 | 0.00E+00 | 21 | RVLKADVETLRAKVKMAEETV |
| 11 | 0.00E+00 | 29 | SPNPSGASGNVANYMGQMAMAMGKLGTLE |
| 12 | 0.00E+00 | 29 | EKQQTLGEMTLEDFLVKAGVVGESFHNNK |
| 13 | 0.00E+00 | 29 | AISNHLQRQGSLTLPRTLSQKTVDEVWKD |
| 14 | 0.00E+00 | 21 | FGEIMDAKKAMPPDKLAELWT |
| 15 | 1.70E-224 | 15 | SHISDIELRMLVDGM |
| 16 | 5.00E-223 | 15 | PYVAMYPHGGIYAHP |
| 17 | 2.50E-214 | 15 | HTPHPYMWGPPQHMM |
| 18 | 0.00E+00 | 50 | FRSWQPSWKSIDLGKQEASSSGVKATPQDKKTNNISVPKSNLGHAEKNHI |
| 19 | 0.00E+00 | 41 | SFTTSLNNQFLHNQRQMSTTPQLNMTTSIRRMPHVSPTINV |
| 20 | 0.00E+00 | 41 | ANQSSASKHNRDGTSMRAAGGSKRSTDGDQDDSNKDYPSSM |
| 21 | 4.50E-203 | 15 | LVRQNSVYSLTLDEV |
| 22 | 4.90E-218 | 21 | MKGIPKRMTGTNMLHTEEGPG |
| 23 | 2.80E-217 | 21 | GNDVTTPRTRHRHSCSVDGST |
| 24 | 6.30E-186 | 21 | WAAMQAYYGPRVAMPPYYNSP |
| 25 | 1.60E-251 | 29 | QVVWLRNENHQLLDKLNNFCETHDKVVQE |

**Supplementary Table 3: Primers used in the qPCR analysis.**

| SN | Primer Name | Sequences |
| --- | --- | --- |
| 1 | Ms_ef1_q1F | CAGAACTGGGTTCTTGATCG |
| 2 | Ms_ef1_q1R | AACCTTTTAGCTGCCTTTCG |
| 3 | MsbZIP79_q1F | CTTGAAGGATGAACGCAAAAC |
| 4 | MsbZIP79_q1R | GTCCCGTTTTCATGTCAGTG |
| 5 | MsbZIP222_q1F | TCATGAAGGATGAACGCAAG |
| 6 | MsbZIP222_q1R | GTCCCGTTTTCATGTCAGTG |
| 7 | MsbZIP31_q1F | TGATGATGGATAGGGAGATGG |
| 8 | MsbZIP31_q1R | CCACCATGCAAACCAAAGAG |
| 9 | MsbZIP109_q1F | GGGGTTTGATACATGATTTGG |
| 10 | MsbZIP109_q1R | CGTGTTGTCACGCTTTATATGTC |
| 11 | MsbZIP117_q1F | GCAACGGAGAAAGAATGAAGG |
| 12 | MsbZIP117_q1R | CATAGGGGTTAGGGGTTTTTG |
| 13 | MsbZIP80_q1F | GCTGATGATGATTCTGACAGTG |
| 14 | MsbZIP80_q1R | CAAGTGGCGAGTGTAAAGAGG |
| 15 | MsbZIP88_q1F | GTTACACATGCTGCTGGTTCTC |
| 16 | MsbZIP88_q1R | GCCACCCATATTATTTGACTGC |

**Supplementary Figure 1: Multiple Sequence Alignment of complete (237) bZIP sequences.** The alignment was performed using MUSCLE 3.8.31 and visualized using Unipro UGENE v.33. A consensus sequence is provided at the top of the figure (in bold). The bars above the consensus sequence present the percentage of consensus amino acid base between the aligned sequences. The ruler below the consensus sequence provides the position of the amino acid base on the aligned sequences. The color changes from light to dark with dark color indicating highly conserved amino acid bases. The consensus sequence at the top indicates the level of conservation with a capital letter indicating high conservation and a small letter with low conservation. The highly conserved bZIP domain (N-x(7)-[RK]-x(9)-L-x(6)-L-x(6)-L), between 590 to 622, is presented in dark color.

**Supplementary Figure 2. Distribution of bZIP genes over the Chromosomes.** The outer circle represents the chromosomes, and all the four chromosomes (.1, .2, .3 and .4) are represented by a single color . The vertical red line indicates the position of the bZIPs in the chromosomes.

**Supplementary Figure 3. Collinearity analysis of *MsbZIP* genes with *Medicago truncatula* and new assembly.** The gray color genes in the background indicate the collinear genes that displayed collinearity with *Medicago truncatula* and with the new assembly. And the red color lines indicate the *MsbZIP* genes that were collinear with *Medicago truncatula* and with the new assembly for the current study.

**Supplementary Figure 4. Expression profile of 152 alfalfa bZIP genes in response to cold stress**. Most of the genes were highly expressed during the initial 2-hour cold treatment and as the duration of treatment increased, the number of highly expressed genes decreased. Few genes that were highly expressed during initial cold treatment of 2 hours were also active during 24 hours of cold treatment. At a duration of 2 hours, 6 hours and 48 hours of cold treatment, different genes were highly expressed suggesting differential gene expression of the bZIP genes. Different groups are represented to the side of the genes by the respective group name along with the color as represented in the phylogenetic tree.

**Supplementary Figure 5. Cis-regulatory elements in the promoter regions of MsbZIP genes.** The numbers inside blocks represent the numbers of cis element identified in the analyzed promoter region of indicated bZIP genes. The cis-element investigated are abscisic acid responsive (ABRE), methyl jasmonate responsive (CGTCA-motif), light inducible G-box motif, low-temperature responsive (LTR), drought responsive (MBS binding site) and defense and stress responsive (TC-rich repeats). The different color represents different groups based on the phylogeny.

**Supplementary Figure 6.** **Representation of motifs identified among 237 bZIP proteins using the “MEME” motif searching tools.** The motifs are indicated by different colors. The alphabet adjacent to the rightmost lines is the name of the group for the sequences and motif. MEME-suite version 5.1.1 was used online for generating the motif location figure. The 25 different motifs are represented by different colors. As the groups are formed based on the similarity of motifs between sequences, the sequences showed high group specificity and were in consensus with the groups from phylogenetic analysis. The sequence for the motif is presented in supplemental information S5. The basic region and the leucine zipper region are represented by motif 1 and motif 6.
